# Genomic Virulence Features of Two Novel Species *Nocardia barduliensis* sp. nov. and *Nocardia gipuzkoensis* sp. nov., Isolated from Patients with Chronic Pulmonary Diseases

**DOI:** 10.3390/microorganisms8101517

**Published:** 2020-10-01

**Authors:** Imen Nouioui, Carlos Cortés-Albayay, Meina Neumann-Schaal, Diego Vicente, Gustavo Cilla, Hans-Peter Klenk, Jose María Marimón, Maria Ercibengoa

**Affiliations:** 1 Leibniz Institute DSMZ–German Collection of Microorganisms and Cell Cultures, 38124 Braunschweig, Germany; meina.neumann-schaal@dsmz.de; 2 Laboratory of Microbial Complexity and Functional Ecology, Antofagasta Institute, University of Antofagasta, Antofagasta 1240000, Chile; c.j.cortesbt@gmail.com; 3 Biodonostia, Infectious Diseases Area, Respiratory Infection and Antimicrobial Resistance Group; Osakidetza Basque Health Service, Donostialdea Integrated Health Organisation, Microbiology Department, 20014 San Sebastian, Spain; diego.vicenteanza@osakidetza.eus (D.V.); carlosgustavosantiago.cillaeguiluz@osakidetza.eus (G.C.); josemaria.marimonortizdez@osakidetza.eus (J.M.M.); 4 School of Natural and Environmental Sciences, Newcastle University, Newcastle upon Tyne NE1 7RU, UK; hans-peter.klenk@newcastle.ac.uk

**Keywords:** actinobacteria, phylogenomics, polyphasic taxonomy, infection

## Abstract

Strains 335427^T^ and 234509^T^, isolated from two 76-year-old patients with chronic pulmonary diseases, were the subject of polyphasic taxonomic studies and comparative genomic analyses for virulence factors. The 16 rRNA gene sequence similarity between strains 335427^T^ and 234509^T^ and their closest phylogenetic neighbors *Nocardia asiatica* NBRC 100129^T^ and *Nocardia abscessus* NBRC 100374^T^ were 99.5% and 100%, respectively. Digital DNA–DNA hybridization values between the aforementioned studied strains were well below the 70% threshold for assigning prokaryotic strains to a novel species. Strains 335427^T^ and 234509^T^ have genome sizes of 8.49 Mpb and 8.07 Mpb, respectively, with G + C content of 68.5%. Isolate 335427^T^ has C_16:0_, C_18:1_ ω_9_c, C_18:0_ and C_18:0_ 10 methyl as major fatty acids (>15%) and mycolic acids formed of 52–54 carbon atoms. However, only C_18:1_ ω_9_c was detected for isolate 234509^T^, which had mycolic acids with 44–56 carbon. Based on phenotypic and genetic data, strains 335427^T^ (DSM 109819^T^ = CECT 9924^T^) and 234509^T^ (DSM 111366^T^ = CECT 30129^T^) merit recognition as novel species, which are named *Nocardia barduliensis* sp. nov. and *Nocardia gipuzkoensis* sp. nov., respectively. All the strains studied had homologous VF-associated genes to those described in *M. tuberculosis*, including experimentally verified virulence genes in humans related to tuberculosis. The *nar*GHIJ (nitrate reduction pathway) and *gvp*AFGOJLMK (gas vesicles) genetic maps of strains 335427^T^, 234509^T^, NBRC 100129^T^ and NBRC 100374^T^ showed the same syntenic block and raise the question of whether their functions are interlinked during the infection of the human host. However, further research is required to decipher the role of the gas vesicle in the pathogenicity mechanism of *Nocardia* spp.

## 1. Introduction

The type genus *Nocardia* [1] of the *Nocardiaceae* family encompasses over 100 species with validly published names, most of which were assigned based on a combination of phenotypic and genetic data. Members of this taxon are environmental saprophytes distributed in aquatic (fresh and saltwater) and terrestrial habitats (soil, dust, feces, vegetation, etc.) in different geographic locations. However, some species, such as *Nocardia brasiliensis* [2] were prevalent in specific areas and climates.

The type species of the genus *Nocardia asteroides* [3], together with more than 40 other species (e.g., *Nocardia abscessus*, *Nocardia africana*, *Nocardia farcinica*, *Nocardia mexicana*, *Nocardia nova*, *Nocardia veterana*, etc.) are opportunistic pathogens for animals and/or humans that cause infection, most notably in immunocompromised diabetics and organ transplant patients, as well as in individuals with connective tissue and lung disorders, chronic alcoholism and corticosteroid therapy [4,5,6]. However, transmission of *Nocardia* infection from human to human has not been reported. Associated with human infections, *Nocardia* spp. have been linked to pulmonary and cutaneous diseases after inhalation and contact with bacteria through a cut or scraped skin. In addition, the infections can be extra-pulmonary causing cerebral abscesses, peritonitis, ocular and catheter-related infections) [6,7]. The disseminated *Nocardia* infections are rarely resulted from cutaneous infections [8].

The clinical symptoms of pulmonary infections are similar to those of tuberculosis with cough, chest pain, fever, night sweats, pneumonia and weight loss [9] but also can be with non-specific symptoms [8]. The most frequently clinical diagnostics are necrotizing pneumonia but empyema, pericarditis, mediastinitis, pleural effusion and superior vena cava obstruction were also reported for local invasive infection [8]. The clinical cutaneous infections are associated to abscess or cellulitis, lymphocutaneous infection and mycetoma [8]. The highest rates of annual nocardiosis incidence were found in developing countries with 1.8 to 4.1% [9] and in the United States of America where approximately 500–1000 new cases of nocardiosis were detected with more male infection than female (3:1) [10,11,12]. Little is known about the pathogenesis of *Nocardia* spp.which are able to enter in the macrophage and inhibit the fusion of phagosomes with lysosomes [13]. Several experimentally verified virulence factors including catalase, superoxide dismutase, phospholipase C, hemolysin (toxic proteins) and protease have been identified in *Nocardia* spp. (*Nocardia brasiliensis, Nocardia farcinica, Nocardia cyriacigeorgica*) [14]. Catalase and superoxide dismutase (SOD) block the function of phagocytes to degrade the bacteria [15] while phospholipase C has the function to destroy the tissue. 

Unfortunately, *Nocardia* spp. have been commonly considered as another member of the human microbiome community, which may explain the limited studies on the infectivity of this taxon. The increased number of *Nocardia* species (>100 species with validly published names [16]), together with their significant clinical impact on human health, call for the application of an outstanding systematic approach, including genome sequence and analysis, to assign each isolate to its corresponding taxonomic rank and evaluate its clinical relevance based on cutting-edge genomic and bioinformatic approaches. 

In this context, to evaluate their pathogenicity, isolates 335427^T^ and 234509^T^, which were recovered from the sputum of patients with pulmonary disease, were the subject of polyphasic taxonomic studies and comparative genomic analyses. Genome-based analyses were performed to identify these isolates’ virulence factors and antibiotic resistance patterns. The resultant data showed that isolates 335427^T^ and 234509^T^ are novel species. The proposed names for the novel species are *Nocardia barduliensis* sp. nov. and *Nocardia gipuzkoensis* sp. nov., respectively. The isolates, along with their closest phylogenetic neighbors (*Nocardia asiatica* NBRC 100129^T^ and *Nocardia abscessus* NBRC 10034^T^), had virulence factors encoding genes homologous to those described for *Mycobacterium tuberculosis*.

## 2. Case Reports

### 2.1. Case 1

The patient was a 76-year-old man with a history of chronic obstructive pulmonary disease, obesity, insulin-dependent type 2 diabetes mellitus, high cholesterol, iron-deficiency anemia, ischemic cardiomyopathy and a gastroduodenal ulcer. On 26 February 2010, he was admitted to the Pneumology Service of Donostia University Hospital with a fever and general discomfort. On examination, the patient had a temperature of 37.8 °C, systolic blood pressure of 160 mmHg and diastolic of 60 mmHg, 32 breaths per minute, heartrate of 84 bpm and an 86% baseline oxygen saturation. The patient was conscious, well-orientated, well-hydrated and had normal skin color. Rales in both pulmonary fields were observed in lung auscultation, though more pronounced on the right. Abdominal and lower extremities examinations were normal. A chest X-ray confirmed pneumonia in the right inferior lobe. Blood analysis showed leukocytosis with neutrophilia. *Streptococcus pneumoniae* and *Legionella pneumophila* urine antigen tests were negative. *Nocardia* spp. (isolate 335427^T^) grew on a sputum specimen which had been obtained on 27 February 2010. There was an absence of other possible bacterial pathogens. The patient was treated with oxygen therapy, nebulized bronchodilators, intravenous steroids and levofloxacin for 10 days. After improving clinically and radiography showing no sign of pneumonia, the patient was discharged on 3 March.

### 2.2. Case 2

The 76-year-old female patient had been diagnosed with bronchiectasis in the middle lobe in 1995 and had subsequently had annual follow-ups though pneumology outpatient care. The patient had no history of drug allergies or other diseases. Flare-ups had become increasingly frequent to the point that they were occurring monthly. Depending mainly on the symptoms and microbiological results, the patient’s condition was managed with microbiological studies of sputum specimens and treated with antimicrobials when necessary. On 8 July 2011, the patient attended her general practitioner for a health check-up after a previous flare-up on 3 May 2011, when her sputum sample (which contained blood) was positive for both *Staphylococcus aureus* and *Pseudomonas aeruginosa*. She was treated with amoxicillin/clavulanic. In July 2011, a sputum specimen was taken and colonies of *S. aureus* and *P. aeruginosa*, along with *Nocardia* spp. were isolated (isolate 234509^T^). In August 2011, the patient went to her general practitioner for her monthly control. At the time of her visit, the patient was quite healthy, with no fever, dyspnea or general impairment. Neither inhaled antimicrobial nor chronic macrolide were administered. In September 2011, the patient started with more abundant expectoration, but did not receive any medical treatment as she was in general good health otherwise.

## 3. Materials and Methods 

### 3.1. Isolation and Phenotypic Characterization 

Strains 335427^T^ and 234509^T^ were isolated from the sputum samples of two patients with pulmonary diseases on BBL MGIT™ (Mycobacteria Growth Indicator Tube) media after 5 days’ incubation at 35 °C as previously described [17,18]. Briefly, sputum samples were decontaminated using the N-acetyl-L-cysteine-sodium hydroxide (NALC-NaOH) method with a final concentration of 1% NaOH (EMD, MA, USA), the pellet neutralized with phosphate buffer (pH 6.8) (EMD, MA, USA), and inoculated into MGIT Tubes (Becton Dickinson and Company, NJ, USA). Two or three drops of the broth from the tubes with positive growth were sub-cultured on 7H11 plates (Seven H11 agar, BBL, MD, USA) and examined daily with a microscope (Zeiss Axio A1, Jena, Germany).

The cultures of the studied isolates were maintained together with their nearest phylogenetic relatives, *N. asiatica* DSM 44668^T^ and *N. abscessus* DSM 44432^T^ (obtained from the German Collection of Microorganisms and Cell Cultures (DSMZ)), on yeast extract-malt extract-agar (International *Streptomyces* project ISP2) medium (BD, MD, USA) [19] for 5–7 days at 28 °C and as suspensions in 35% (*v/v*) glycerol at −80°C. Cultural properties for both isolates were performed on the following agar media after 7 days of incubation at 28°C: ISP2, GYM (DSMZ medium 65), Middlebrook 7H9 (MB7H9, BD, MD, USA [20,21]), nutrient agar (NA, [22]), peptone-meat extract-glucose agar (DSMZ medium 250) and tryptic soy agar (TSA, BBL, BD, MD, USA [23]), GPHF medium (DSMZ 553). In addition, the growth of isolates 335427^T^ and 234509^T^ were tested in the presence of a wide temperature range: 4°C, 10°C, 15°C, 25°C, 28°C, 37°C, 42°C and 45°C, as well as under anaerobic conditions using anaerobic atmosphere generation bags (Sigma-Aldrich 68061, St. Louis, MO, USA).

Biochemical properties of the studied isolates and their relatives *N. asiatica* DSM 44668^T^ and *N. abscessus* DSM 44432^T^ were recorded from arylsulfatase after 3 and 14 days [24], as well as the results of the tests for reducing potassium tellurite [25,26]. An inoculum of 100 µl of a homogeneous bacterial suspension at mid-logarithmic growth phase (OD_600_ = 0.8) were used for these assays. The studied strains’ ability to metabolize a broad range of carbon and nitrogen sources, as well as to grow in the presence of inhibitory compounds and different concentrations of sodium chloride, were carried out using GENIII microplates in an Omnilog devise (Biolog Inc., Hayward, CA, USA). The plates were inoculated with bacterial suspension (75% transmittance) and prepared in an inoculating fluid C (IFC) solution provided by the manufacturer. The resultant data were analyzed using OPM package version 1.3.36 [27]. All the tests cited above were performed in duplicate.

Antimicrobial susceptibility tests against amikacin, amoxicillin-clavulanic acid, cefepime, ceftriaxone ciprofloxacin, clarithromycin, doxycycline, imipenem, levofloxacin, linezolid, minocycline, tigecycline, tobramycin and trimethoprim-sulfamethoxazole were performed for isolates 335427^T^ and 234509^T^ with the broth microdilution method using Sensititre microtiter trays (Thermo Fisher, Inc., West Sussex, UK), according to the Clinical and Laboratory Standards Institute (CLSI) guidelines and interpretative criteria for *Nocardia* [28]. Penicillin and amoxicillin minimal inhibitory concentrations (MICs) were determined by the E-test method [29] using commercially manufactured strips (bioMérieux, Marcy-l’Étoile, France).

Chemotaxonomic analyses of isolates 335427^T^ and 234509^T^ together with their closest neighbors, *N. asiatica* DSM 44668^T^ and *N. abscessus* DSM 44432^T^, were performed using freeze-dried cells obtained from 7-day-old cultures in shake flasks (200 rpm) of ISP2 medium and incubated at 28 °C. Standard chromatographic procedures were carried out to determine the polar lipid profile; the extracts were prepared using the integrated method of Mininkin et al. [28]. Cellular fatty acid and mycolic acid patterns were identified following the protocols of Miller [30] and Minnikin and Goodfellow [31], respectively. Fatty acids were identified using Standard Microbial Identification (MIDI) system version 4.5 and the ACTIN6 database (Sasser) [32]. Fatty acid and mycolic acid profiles were determined using gas chromatography (Agilent Technologies 6890N, Santa Clara, CA, USA).

### 3.2. Whole Genome Sequencing

Genomic DNA of isolates 335427^T^ and 234509^T^ were extracted, purified and quantified following the protocol adopted by MicrobesNG service in Birmingham (UK). Genomic DNA libraries were performed using Nextera XT library Prep Kit (Illumina, San Diego, CA, USA), according to the manufacturer’s protocol with some modifications (two nanograms of DNA were used and PCR elongation time was increased to 1 min). Preparing and quantifying the DNA were performed on a Hamilton Microlab STAR automated liquid handling system. An Illumina HiSeq with a 250bp paired end protocol was used for genome sequencing (MicrobesNG, Birmingham, UK). Trimmomatic 0.30 with a sliding window quality cut-off of Q15 [33] was used as the read trimming tool. SPAdes version 3.7 [34] and Prokka 1.11 [35] were used for De Novo assembly and to annotate the contigs, respectively. The draft genome sequence of isolates 335427^T^ and 234509^T^ were annotated and affiliated to several functional classes in the RAST-SEED webserver [36]. A digital DNA–DNA hybridization (dDDH) was performed between the draft genome sequences of the studied isolates and their close phylogenetic neighbors with the 16S rRNA gene sequence similarities value above the 98.7% threshold for assigning prokaryotic strain to a new species [37]. The dDDH was performed using the Genome-to-Genome distance calculator (GGDC) web server and the recommended formula 2 of Meier-Kolthoff et al. [38]. 

### 3.3. Identification and Phylogenetic Studies

Full length of the 16S rRNA gene sequences of isolates 335427^T^ (1525 pb: accession number MT472102) and 234509^T^ (1525 bp; accession number MT704612), as well as partial sequences of *hsp*65, *sec*A1, *gyr*B, and *rpo*B genes were extracted from the draft genome sequences which were submitted in GenBank under accession numbers JABLTE000000000 and JACBNG000000000, respectively. However, the 16S rRNA gene sequences of the references strains were retrieved from EzBioCloud database [39]. The 16S rRNA gene pairwise sequence similarity was carried out through the GGDC web server (http://ggdc.dsmz.de/) [38,40] following the DSMZ phylogenomics pipeline [41]. The similarity values between the four housekeeping gene sequences of both isolates and their nearest neighbors were determined using BLASTp and the NCBI database [42]. Phylogenetic trees based on single gene and genome sequences were inferred from the Type Strain Genome Server (TYG), a high-throughput web server available at GGDC web server [43].

### 3.4. Virulence Factors

The draft genome sequences of strains 335427^T^, 234509^T^, NBRC 100129^T^ and NBRC 100374^T^ were interrogated for the virulence factors using the Virulence Factors (VFs) of Pathogenic Bacteria web server [44] including *Mycobacterium tuberculosis* as the reference genome. The predicted coding sequences (CDS) were identified using the GLIMMER3 system (system for finding genes in microbial DNA) prior to using the VFanalyzer tool. We then screened the pathogenic genes that are related to amino acid, purine, cholesterol, lipid and fatty acid metabolism; copper, iron and magnesium uptake; cell surface components; mammalian cell entry (mce); anti-apoptosis mechanisms; phagosome arresting; stress adaptation and secretion system. 

Based on the concept that the “true or specialized virulence gene” products are involved in interactions with the host (pili, antigen) and are directly responsible for the pathological damage caused during infection [45], the genomes of isolates 335427^T^ and 234509^T^ were subjected to screening for VFs associated with host–pathogen interactions experimentally verified to cause human diseases. In this context, the Pathogen-Host Interaction database (PHI-base version 4.9) (http://www.phi-base.org/) was used. The later houses genes experimentally proved to affect the outcome of pathogen host interaction [46]. The selection of VF–genes from PHI-base was restricted to the following criteria:

- Pathogen species: *Mycobacterium tuberculosis*
- Disease: tuberculosis
- Host species: *Homo sapiens* (related to human) and *Mus musculus* (house mouse)
- Experimental technique: partial and full gene deletions, complementation and disruption.
- Mutant phenotype: increased virulence (hypervirulence); loss of pathogenicity; reduced virulence.

The presence of the selected VFs was checked manually in the genome sequences of isolates 335427^T^ and 234509^T^ using RAST-SEED webserver. BLAST of the amino acid sequences of the VF genes was performed using Basic Local Alignment Tool (BLASTp). The conserved protein domains for some amino acid sequences were confirmed after comparing them with those available in the Conserved Domains Database (CDD) of NCBI [47].

The PathogenFinder server [48] was used to estimate the probability of isolates 335427^T^ and 234509^T^ being human pathogens based on a comparative genomic approach with the genome sequences of well-known pathogenic actinobacterial strains.

### 3.5. Antimicrobial Gene Resistance and Toxic Compound Tolerance

Genome sequences of isolates 335427^T^ and 234509^T^ were screened for the presence of AMR genes using the Comprehensive Antibiotic Resistance Database (CARD version 3.0.8) and Resistance Gene Identifier (RGI 5.1.0) software which are based on the bioinformatics pipeline described by Alcock et al. [49]. 

The tolerance of these isolates to toxic compounds was based on an in silico genome analysis using Rapid Annotation Subsystem Technology (RAST) [35] and the SEED servers [50]. 

### 3.6. Comparative Genomic of VF-Associated Genes

Comparative genomics were performed for the pathogenicity genes between the annotated draft genome sequences of isolates 335427^T^ and 234509^T^ with those of their closest relatives *N. asiatica* NBRC 100129^T^ and *N. abscessus* NBRC 100374^T^. The predicted genes were manually curated and annotated using ARTEMIS [51]. Whole-genome comparisons were performed using BLASTN and visualized using the BLAST Ring Image Generator (BRIG) V0.95 [52] and EasyFig V2.2 [53].

## 4. Results and Discussion

### 4.1. Phenotypic Characterization

Isolates 335427^T^ and 234509^T^ formed rough, white-pinkish aerial mycelia on GYM, ISP2, MB7H9 and TSA agar media after 7 days of incubation at 28 °C, while the colonies of isolate 335427^T^ had a pale yellow-orange color on DSMZ 250 medium. Poor growth was detected on NA medium for both strains. Isolates 335427^T^ and 234509^T^ were able to grow at 25 °C, 28 °C, 37 °C and 42 °C, but not in the presence of any media incubated at 4 °C, 15 °C, 20 °C, and 45 °C. Optimal growth of isolate 335427^T^ was shown on ISP2, GYM, MB7H9 and TSA media at pH 7 after 5–7 days at 28°C and 37°C; there was no growth detected under anaerobic conditions for either isolate. The same results were obtained for isolate 234509^T^ with the exception that it displayed good growth in the DSMZ 250 medium.

Members of the genus *Nocardia* are characterized by the presence of arabinose, galactose and *meso*-2,6-diaminopimelic acid, as whole-cell hydrolysates with A1γ type peptidoglycan and MK-8(H4, ω-cyclo) as the predominant isoprenologue [54,55]. The polar lipid profile of *Nocardia* strains contains diphosphatidylglycerol, phosphatidylethanolamine, phosphatidylinositol and phosphatidylinositol mannosides, in addition to the nocobactins, which are characteristic lipids for this taxon [54,55]. Fatty acid patterns had significant amounts of straight-chain, saturated, unsaturated and 10-methyl (tuberculostearic) and mycolic acids with 46–64 carbon atoms [55]. The chemotaxonomic features of the studied isolates are in line with the genus *Nocardia*. The polar lipid profile of isolate 335427^T^ contained diposphatidylglycerol (DPG), phosphatidylethanolamine (PE), two unidentified glycophospholipids (GPL1-2) and four unidentified lipids (L1-4), while its closest relative *N. asiatica* DSM 44668^T^ had aminoglycophospholipid (AGPL) (Figure S1). Major fatty acids (>15%) of strain 335427^T^ were C_16:0_, C_18:1_ ω_9_c, C_18:0_ and C_18:0_ 10 methyl, as is the case for its relative, strain DSM 44668^T^ (Table S1). Isolate 335427^T^ can be distinguished from its close phylogenetic relative, *N. asiatica* DSM 44668^T^, by its mycolic acids profiles that contain 52–54 carbon atoms as the major ones, while *N. asiatica* has 50–52 carbon atoms.

Isolate 234509^T^ can be distinguished from *N. abscessus* DSM 44432^T^ by its fatty acid patterns. The major fatty acids of isolate 234509^T^ were C_18:1_ ω_9_c (70%), while strain DSM 44432^T^ had C_16:0_ (33.8%) and C_18:1_ ω_9_c (24%) (Table S1). Isolate 234509^T^ had mycolic acid with 44–56 carbon atoms, unlike the type strain of *N. abscessus* species which had 46–56.

Isolates 335427^T^ and 234509^T^ were distinguishable from one another and from their closest relatives based on their ability to metabolize carbon sources, amino acids and organic acids and to grow in the presence of inhibitory compounds (Table 1). Identical results were obtained for all the duplicated tests. All the studied strains 335427^T^, 234509^T^, DSM 44668^T^ and DSM 44432^T^ were unable to produce arylsulfatase within 3 and 14 days and were not inhibited by potassium tellurite.

All strains were able to metabolize sucrose and glycerol (carbon sources); L-glutamic acid (amino acid); acetic acid, citric acid; β-hydroxy-butyric acid; α-ketoglutaric acid; L-malic acid and propionic acid (organic acids); to grow in the presence of aztreonam; lincomycin; nalidixic acid; potassium tellurite; rifamycin SV; troleandomycin and Tween 40 (Inhibitory compounds); and at 1% (w/v) NaCl and pH 5-6. In contrast, none of the strains used α-D-lactose; D-mannitol; D-raffinose; D-sorbitol and stachyose (carbon sources); L-alanine; L-arginine; D-aspartic acid; L-aspartic acid; D-serine #1; gly-pro and L-pyroglutamic acid (amino acids); γ-amino-n-butyric acid; L-galactonic acid-γ-lacton and quinic acid (organic acids); and were unable to grow in the presence of guanidine hydrochloride; fusidic acid; minocycline; niaproof; sodium formate; tetrazolium blue; 4–8% NaCl and pH 5.0.

### 4.2. Molecular Characterization and Genome-Based Taxonomy

We targeted four housekeeping genes that are known to be valuable in nocardial systematics. The blast of the partial sequences of the *hsp*65, *sec*A1, *gyr*B, and *rpo*B genes of isolates 335427^T^ and 234509^T^ confirmed their assignment to the genus *Nocardia* (Table 2).

The 16S rRNA gene sequence similarity values between isolate 335427^T^ and its relatives *N. asiatica* NBRC 100129^T^, *N. arthritidis* NBRC 100137^T^ and *N. abscessus* IMMIB D-1592^T^ were 99.5%, 99.0% and 98.9%, respectively. However, the similarity value was 100% between isolate 234509^T^ and *N. abscessus* IMMIB D-1592^T^ and ranged from 99.0–98.9% with *Nocardia exalbida* NBRC 100660^T^, *N. asiatica* NBRC 100129^T^ and *N. asteroides* NBRC 1553^T^, respectively (Table 2).

**Figure 1:**
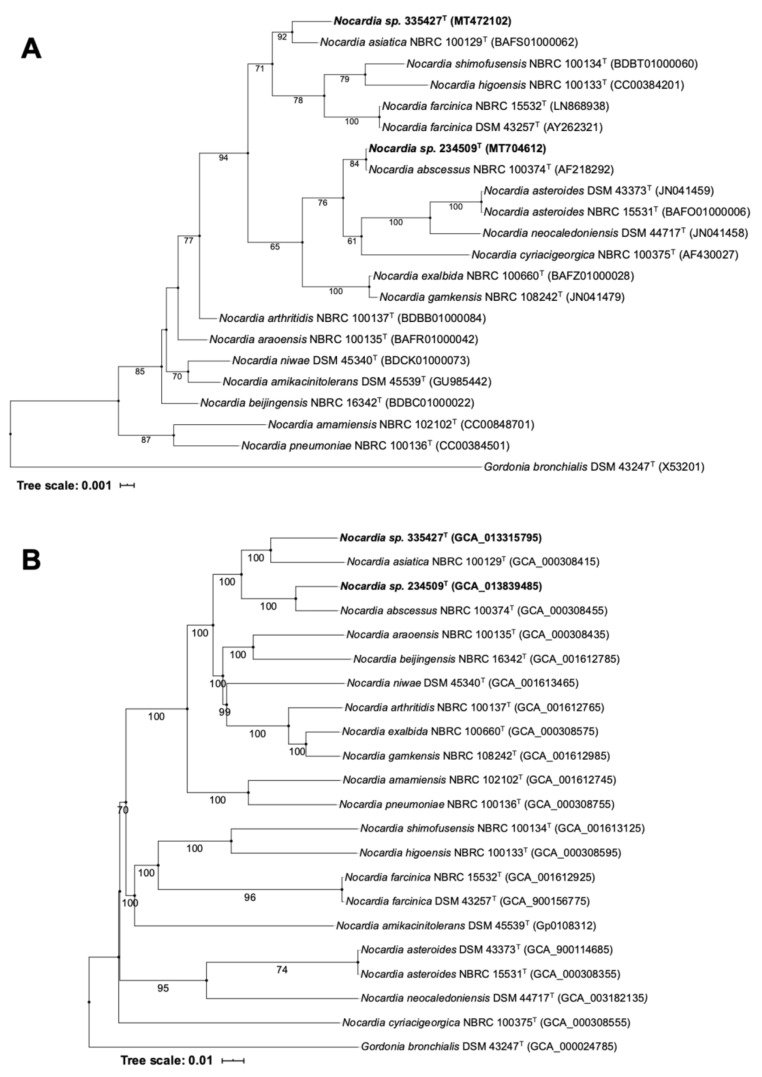
Phylogenetic trees based on 16S rRNA gene (**A**) and genome sequences (**B**) showing the phylogenetic position of isolates 335427^T^ and 234509^T^ within the evolutionary radiation of the genus *Nocardia* and their relationship with one another and with their closest phylogenetic relatives.

In the 16S rRNA phylogenetic tree (Figure 1A), isolate 335427^T^ along with *N. asiatica* NBRC 100129^T^ formed a poorly supported subclade located adjacent the one housing the *Nocardia higoensis*, *N. shimofusensis* and *N. farcinica* species. However, isolate 234509^T^ appeared in the same branch as *N. abscessus* NBRC 100374^T^ and was loosely associated to a subclade containing the *N. asteroides*, *N. neocaledoniensis* and *N. cyriacigeorgica* species. The 16S rRNA gene phylogenetic tree’s topology is contradictory to the genome-based phylogeny (Figure 1B) in which isolates 335427^T^ and 234509^T^ formed well-supported subclades with *N. asiatica* NBRC 100129^T^ and *N. abscessus* NBRC 100374^T^, respectively. The clade housing these strains was next to the one which encompasses mainly clinical *Nocardia* species which are classified as risk group 2 bacteria (RG2): *Nocardia araoensis* NBRC 100135^T^ (isolated from human) [56], *Nocardia beijingensis* NBRC 16342^T^ (isolated from mud but members of this species were also found in human infectious samples) [57,58], *Nocardia niwae* DSM 45340^T^ (isolated from lung biopsy) [59], *Nocardia arthritidis* NBRC 100137^T^ (isolated from human sputum) [60], *Nocardia exalbida* NBRC 100660^T^ *(*isolated from the bronchoalveolar lavage of a patient with lung nocardiosis) [61] and *Nocardia gamkensis* NBRC 108242^T^ (isolated from soil) [62]. It has been reported that all these species were involved in human disease (except *N. gamkensis*) and they clustered together based on different phylogenetic studies [63,64]. These phylogenetic results highlighted the pathogenicity potential that both isolates may have.

Draft genome sequence is a powerful data source for a trustworthy classification into the corresponding taxonomic rank and for understanding the biology of these clinical isolates. In this context, the dDDH between the draft genome sequences of isolates 335427^T^ and 234509^T^ and their phylogenetic neighbors were well below the established threshold of 70%, designed for prokaryotic species’ affiliation [65] (Table 2). The dDDH value between both isolates was 44.2% (Table 2).

Isolate 335427^T^ has a genome size of 8.49 Mpb with N50 contig length 65567, L50 contig count 42, 8527 coding sequences, 57 RNAs, 312 contigs and an in silico G + C content of 68.5%. These genomic features are in line with those of the closest neighbor *N. asiatica* (genome size of 8.46 Mb, 50 RNAs, and G + C content 68.4%). Isolate 234509^T^ has a genome size of 8.07 Mpb with N50 contig length 108726, L50 contig count 62, 57 RNAs, 7974 coding sequences and an in silico G + C content of 68.5%; genomic traits that are similar to its closest neighbor *N. abscessus* NBRC 100374^T^ (8.41 Mpb, 54 RNAs, G + C content 68.2%).

### 4.3. Virulence Factors

Despite the increasing numbers of molecular studies related to the pathogenic mechanisms involved in nocardial infection, little is known about the virulence factors (VFs) of this taxon. The well-known VFs experimentally approved in *Nocardia* strains are catalase, cell-wall lipids, superoxide dismutase, hydrolases, lipases and proteases [66].

**Figure 2:**
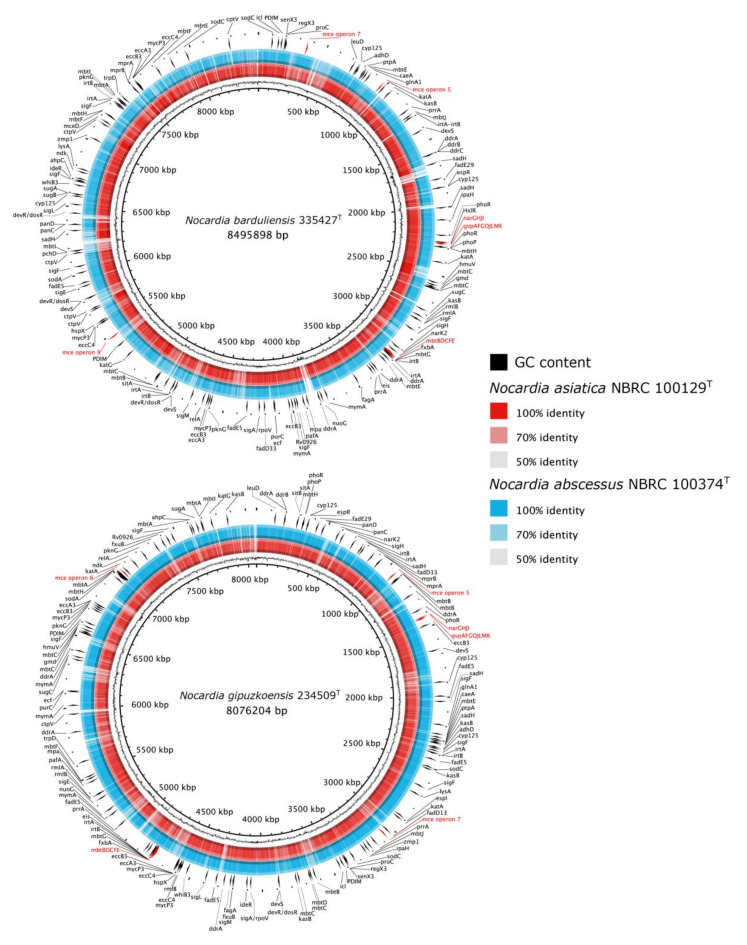
Comparative genomic map of the pathogenicity genes of isolates 335427^T^ and 234509^T^ genomes and their closest type strains. The circadian maps represent a BLASTN comparison of the genomes of isolates 335427^T^ and 234509^T^ (black inner rings) and its GC content (black frequency ring) with the genomes *N. asiatica* NBRC 100129^T^ (red ring) and *N. abscessus* NBRC 100374^T^ (blue ring). The pathogenicity genes are annotated with black labelled arrows and labels in red. The legends show the percentages of identity of genome alignment.

*N. asiatica* DSM 44668^T^ is one of the species associated with lung and central nervous system (CNS) infections that lead to brain abscesses. This species is closely related to *N. abscessus*, which was isolated from an abscess of a patient with knee endoprosthesis [67]. The close phylogenetic relationship between these two species and the studied isolates call for a closer look at the VF-encoding genes in their genome sequences. For this reason, an in silico genome screening for VFs was carried out and the resultant data showed that all the strains share almost the same set of well-known VF-encoding genes involved in amino acid, purine (e.g., *pur*C, *gln*A1), lipid and fatty acid metabolism (e.g., *icl*); catabolism of cholesterol (e.g., *cyp*125, *fad*E29); and expression, processing or secretion of virulence factors (e.g., *hsp*X, *eis*, *pkn*G) (Figure 2). Most of these genes play an important role in bacteria’s survival and some of them are considered as housekeeping genes that are well-conserved not only in bacteria, but also in fungi, plants and protists. However, the true virulence genes, the products of which interact with the host, and are directly involved in the pathological damage inflicted during infection [44], were also found in all the strains included in this present report (Figure 2). Genes homologous to those of *M. tuberculosis* H37Rv with functions related to the secretion system, phagosome arresting, and anti-apoptosis biosynthesis factors were also detected in all the strains (Figure 2). The genome of all the studied *Nocardia* strains have genes associated with intracellular survival protein (*eis*) whose product is related to the intracellular survival of *Mycobacterium* in macrophage cell lines [68]. All the strains’ genomes contained nucleoside diphosphate kinase gene (*ndk*) known for its role in protecting against reactive oxygen species [69]. In addition, the genomes of strains 335427^T^, 234509^T^ and DSM 44668^T^ were found to have the *nuo*G gene which encodes for the subunit of the type I NADH-dehydrogenase that inhibits the apoptosis of infected cells [70] (Figure 2).

All the studied strains seem to have the same genetic potential for stress adaptation (Figure 2) such as catalase (*kat*A), catalase peroxidase (*kat*G) and alkylhydroperoxide reductase (*ahp*C). The *ahp*C gene plays a role in the resistance to peroxynitrite produced by macrophage as host defense against bacteria [71].

The genome of both isolates 335427^T^ and 234509^T^ housed experimentally verified virulence genes on human and *Mus musculus* that are involved in tuberculosis disease as shown in Table S2.

### 4.4. Comparative Genomic of VF-Encoding Genes

The genomes of isolates 335427^T^ and 234509^T^ contained 167 and 154 genes, respectively, encoding for virulence factor proteins, including those involved in adherence to the host cells (Figure 2). In this context, the mce proteins which were found on the mycobacterial cell surface and found to play a role in invading the host cell during infection were also detected in *Nocardia* species [72]. Isolate 335427^T^ presented the complete mce operons 5, 7 and 9, while isolate 234509^T^ had operons 7 and 6. All the operons showed identical genes: two inner membrane permeases YrbEa and YrbEb and six mceA-F integral membrane proteins [72,73]. In contrast, the closest phylogenetic species *N. asiatica* NBRC 100129^T^ was devoid of any mce operon on its draft genome sequence unlike *N. abscessus* NBRC 100374^T^ which had operons 5, 6 and 7.

**Figure 3:**
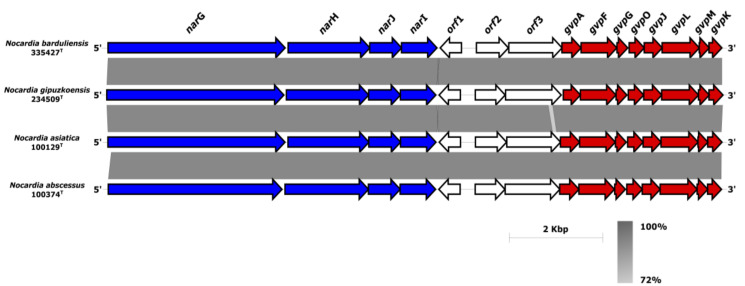
Synteny of the *nar* and *gvp* gene clusters sub-region in isolates 335427^T^ and 234509^T^ and their closest relatives. All the genes present on these clusters showed identity values over 72% between isolates 3355612^T^ and 234509^T^ and the closest neighbors.

Moreover, another operon named *nar*GHIJ was detected in the genomes of isolates 335427^T^ and 234509^T^ and their closest relatives (Figure 3). The *nar*GHIJ operon is involved in the nitrate reduction pathway and its products are involved in the survival of pathogenic bacteria during low-oxygen-levels-mediated dormancy in human infections [74,75]. The *nar*G, *nar*H, *nar*I *nar*J genes encoded for nitrate reductase alpha (COG5013), beta (COG1140), gamma (COG2181) and delta subunits (COG2180), respectively (Figure 3). Next to the three ORFs downstream of the *nar*GHIJ gene cluster, a well-organized *gvp* gene cluster formed with *gvp*AFGOJLMK (Figure 3) was detected. The *gvp* gene cluster encoded for gas vesicle proteins provide buoyancy and enable the cells to seek optimal growth conditions in response to growth requirements depletion [76]. The gas vesicles were considered to be flotation devices for cyanobacteria and halophilic archaea that help them adapt to the light and to look for nutrients in the water. They have also been detected in soil-living actinomycetes such as *Frankia*, *Streptomyces* and *Rhodococcus* [77]. However, Han et al. [78] found that only the *Nocardia* species originated from human clinical samples (*N. concava*, *N. inohanensis*, *N. niigatensis*, and *N. yamanashiensis*), harbored in their genomes the *gvp* genes [78]. The synteny of *gvp* and *nar* clusters (Figure 3) raises the question whether the nitrate reduction pathway and the gas vesicle formation are co-expressed during the infectivity process. The gas vesicles might serve as motility organelles allowing *Nocardia* to move and propagate within the host in response to stressors as has been described in another taxa [76,77]. Further study is required to decipher the role of these two clusters in clinical *Nocardia* isolates.

### 4.5. Estimation of Being Human Pathogen

Comparing the genome of isolate 335427^T^ with actinobacterial genomes available in PathogenFinder database server showed that it has a 66.5% probability of being a human pathogen. Similarity values of 88.7–96.7% were estimated between the genome of the studied isolate and the eight pathogenic gene families that belong to *Nocardia farcinica* IFM 10152, *Gordonia bronchialis* DSM 43247^T^ and *Corynebacterium* spp. However, isolate 234509^T^ showed a higher probability of 75.4% of being a human pathogen than isolate 335427^T^ (66.5%) and its closest relative, *N. abscessus* NBRC 100374^T^ (63.6%). Similarity values ranged between 89.5–99.0%, with protein sequences of the eight pathogenic family of *Nocardia farcinica* IFM 10152, which is the causative agent of bovine farcy in animals and pulmonary nocardiosis in humans [79].

### 4.6. Antimicrobial Gene Resistance and Toxic Compound Tolerance

Isolate 335427^T^ was susceptible to all the tested antibiotics except for amoxicillin-clavulanic acid (Table 3) while isolate 234509^T^ was non-susceptible to levofloxacin and ciprofloxacin, but was sensitive to amoxicillin/clavulanic acid, ampicillin, cefazolin, ceftazidime, amikacin, cefotaxime, ceftriaxone, imipenem, linezolid, tetracycline and trimethoprim-sulfamethoxazole (Table 3). This antimicrobial susceptibility profile of isolate 234509^T^ is similar to pattern XI of *Nocardia amikacinitolerans* (RG2, isolated from human eye) and *N. beijingensis* (resistant or intermediate to ciprofloxacin, azithromycin, clarithromycin, clindamycin, ethambutol, isoniazid, levofloxacin, ofloxacin and streptomycin. Susceptible to amoxicillin-clavulanic acid, ampicillin, cefepime, cefotaxime, ceftriaxone, imipenem, linezolid, meropenem and trimethoprim-sulfamethoxazole) [80]. Reann et al. [58] have reported human infection associated to pulmonary abscess caused by *N. beijingensis.*

The genome sequences of strains 335427^T^ and DSM 44668^T^ housed genes related to copper homeostasis and tolerance, cobalt-zinc-cadmium and fluoroquinolones resistance, and mercury reduction. The same results were obtained for strain 234509^T^ and its closest neighbor, *N. abscessus* NBRC 100374^T^, with additional genes which are associated to arsenic resistance.

CARD encompasses known AMR determinants (i.e., acquired resistance genes, resistant mutations of housekeeping genes, efflux overexpression, etc.), drug targets, antibiotic molecules and drug classes, and the molecular mechanisms of resistance.

The in silico screening of AMR genes in the genome sequences of isolates 335427^T^ and 234509^T^ revealed 2–4 AMR gene families as shown in Table 4. It has been shown that expression of the antibiotic resistance genes is significantly higher in vivo than in vitro [81,82] which explains the discrepancies between the in silico and the in vitro antibiotic susceptibility results in this report.

### 4.7. Description of Nocardia barduliensis sp. nov.

Bar.du.li.en’sis—N.L. masc. / fem. Adj. *barduliensis*, referring to Bardulia, ancient name of the region of Spain where the patient from whom the strain was isolated lived.

A gram-positive, aerobic, non-motile actinobacterium with white pinkish aerial mycelium on GYM, ISP2 and TSA agar media, at pH 7, after seven days of incubation at 28 °C and 37 °C. The strain was unable to produce arylsulfatase within 3 and 14 days and its growth was not inhibited by potassium tellurite.

The polar lipid profile contained diposphatidylglycerol, phosphatidylethanolamine, two unidentified glycophospholipids (GPL1-2) and four unidentified lipids (L1-4). The fatty acids pattern consisted of C_16:0_, C_18:1_ ω_9_c, C_18:0_, C_18:0_ 10-methyl and summed feature 3 (C_16:1_ ω_7_c/C_16:1_ ω_6_c). It has mycolic acid with 52–54 carbon chain.

The type strain 335427^T^ (DSM 109819^T^ = CECT 9924^T^) was isolated from the sputum of a 76-year-old male patient. The genome size was 8.49 Mb with an in silico G + C content of 68.5 mol%. The GenBank accession number of the 16S rRNA gene sequence is MT472102. The Whole Genome Shotgun project has been deposited at DDBJ/ENA/GenBank under the accession JABLTE000000000. The version described in this paper is version JABLTE000000000.1.

### 4.8. Description of Nocardia gipuzkoensis sp. nov.

Gi.puz.ko.en’sis—N.L. masc. / fem. adj. *gipuzkoensis*, referring to the province of Gipuzkoa, where the hospital is located and where the patient from whom the strain was isolated was born.

A gram-positive, aerobic, non-motile actinobacterium with white-pinkish aerial mycelium on DSMZ 250, GYM, ISP2, MB7H9 and TSA agar media, at pH 7, after 7 days of incubation at 28 °C and 37 °C. The strain was unable to produce arylsulfatase within 3 and 14 days and its growth was not inhibited by potassium tellurite.

The fatty acid pattern consisted of C_16:0_, C_16:1_ ω_9_c and C_18:1_ ω_9_c. It has mycolic acid with 44–56 carbon chain.

The type strain 234509^T^ (DSM 111366^T^ = CECT 30129^T^) was isolated from the sputum of a 76-year-old female patient. The genome size was 8.07 Mb with an in silico G + C content of 68.5 mol%. The GenBank accession number of the 16S rRNA gene sequence is MT704612. The Whole Genome Shotgun project has been deposited at DDBJ/ENA/GenBank under the accession JACBNG000000000. The version described in this paper is version JACBNG000000000.1.

## 5. Conclusions

Phenotypic, phylogenetic and genomic data distinguished isolates 335427^T^ and 234509^T^ from each other as well as from their closest relatives, *N. asiatica* and *N. abscessus* species. Therefore, isolates 335427^T^ and 234509^T^ merited the assignment as novel species with the proposed names *Nocardia barduliensis* sp. nov. and *Nocardia gipuzkoensis* sp. nov., respectively. The close phylogenetic relationships between the isolates and the *Nocardia* species involved in human diseases are coherent with the comparative genomic-based assessment. The presence in the genome sequences of both isolates of homologous VF genes to those identified in *M. tuberculosis* is in line with their clinical forms of pulmonary infection which cause pneumonia-like symptoms. These results were coherent with the similarity between the antimicrobial resistance profiles of isolate 234509^T^ and *N. beijingensis* that was reported in human pulmonary abscess. The presence of the experimentally proved VF genes associated to tuberculosis in the genome sequences of these *Nocardia* isolates calls microbiologists and clinicians to carefully treat *Nocardia* infection. Genomic information showed that strains 335427^T^, 234509^T^, *N. asiatica* NBRC 100129^T^ and *N. abscessus* NBRC 100374^T^ shared several genes required to invade a host cell and cause damage, and raise questions about the role of gas vesicles in the *Nocardia* pathogenicity mechanism which is still not fully understood at the present time.

## Figures and Tables

**Table 1:** Phenotypic features that distinguish strains 335427^T^ and 234509^T^ from their nearest phylogenetic neighbors *N. asiatica* DSM 44668T and *N. abscessus* DSM 44432^T^.

Characteristics	Isolate 335427^T^	*N. asiatica* DSM 44668^T^	Isolate 234509^T^	*N. abscessus* DSM 44432^T^
**Carbon source utilization**				
β-Gentiobiose and L-Fucose	+	+	-	-
β-Methyl-D-Glucoside	+	w	w	+
Dextrin	-	+	-	+
D-Arabitol	w	+	-	-
D-Cellobiose	-	-	-	w
D-Fructose, D-Fructose-6-Phosphate, *N*-Acetyl-β-D-Mannosamine and L-Rhamnose	+	+	-	+
D-Fucose; D-Galactose, D-Mannose, Inosine, myo-Inositol, 3-O-Methyl-D-Glucose and *N*-Acetyl-D-Galactosamine	+	-	-	-
D-Galacturonic Acid and Glucuronamide	-	w	-	-
D-Glucose and Turanose	w	+	+	+
D-Glucose-6-Phosphate	+	-	+	+
D-Maltose	w	-	+	+
D-Melibiose	w	+	-	+
D-Saccharic Acid and *N*-Acetyl-D-Glucosamine	+	w	+	+
D-Salicin	w	w	+	+
D-Trehalose	-	-	+	w
Gelatin	-	+	-	-
*N*-Acetyl-Neuraminic Acid	+	w	-	-
Pectin	+	w	-	w
**Aminoacids**				
L-Histidine	+	+	-	+
L-Serine	w	-	-	+
**Organic acids**				
Acetoacetic Acid	+	w	+	+
α-Hydroxy-Butyric Acid	+	+	-	+
α-Keto-Butyric Acid	+	-	-	+
Bromo-Succinic Acid	-	-	+	+
Butyric Acid	w	-	w	+
D-Gluconic Acid	w	+	-	-
D-Glucuronic Acid	+	w	+	-
D-Lactic Acid Methyl Ester	+	-	w	-
D-Malic Acid	+	+	+	w
L-Lactic Acid	+	+	w	-
Mucic Acid	+	+	-	-
Methyl Pyruvate	w	-	+	+
*p*-Hydroxy-Phenylacetic Acid	-	-	-	+
**Inhibitory compounds**				
Lithium Chloride	-	-	-	w
Sodium Bromate	-	-	-	+
Tetrazolium Violet	w	-	+	-
Vancomycin	-	-	-	+
1% Sodium Lactate	w	-	+	+

**Symbols**: +, Positive reaction; w, weak reaction; -, Negative reaction.

**Table 2:** 16S rRNA and housekeeping genes similarity as well as the digital DNA–DNA hybridization (dDDH) values (℅) between isolates 335427^T^ and 234509^T^ and their closest phylogenetic relatives.

		Gene Identity (%)					
Isolate	Hit Taxon	16S rRNA	*hsp*65	*sec*A	*gyr*B	*rpo*B	dDDH
335427^T^							
	Isolate 234509^T^	99.0	98.7	97.1	92.9	99.8	44.2
	*N. asiatica*	99.5	98.1	98.5	94.4	99.7	53.5
	*N. arthritidis*	99.0	96.2	97.0	92.8	96.0	36.6
	*N. abscessus*	98.9	97.5	97.9	93.1	99.4	44.6
	*N. farcinica*	98.8	92.9	88.5	91.9	97.2	23.3
	*N. kroppenstedtii*	98.7	-	-	-	-	N/A
	*N. araoensis*	98.6	98.3	97.0		98.5	35.7
	*N. beijingensis*	98.4	97.9	97.6	93.5	98.2	35.0
234509^T^							
	Isolate 335427^T^	99.0	98.7	97.1	92.9	99.8	44.2
	*N. abscessus*	100.0	98.5	98.5	99.8	99.4	67.9
	*N. asiatica*	99.0	98.3	98.2	97.9	99.2	43.0
	*N. exalbida*	99.0	98.8	96.4	97.9	99.0	37.6
	*N. cyriacigeorgica*	98.9	96.3	87.9	93.3	97.5	22.9
	*N. shimofusensis*	98.9	96.8	85.9	92.8	97.6	22.5
	*N. asteroides*	98.7	95.5	98.5	92.7	92.7	22.1
	*N. arthritidis*	98.5	98.5	96.5	91.9	99.1	37.6
	*N. neocaledoniensis*	98.3	95.9	86.5	92.6	96.4	22.1

N/A = not applicable due to the absence of genome sequence.

**Table 3:** Antimicrobial MIC of isolates 234509^T^ and 335427^T^ against 16 antibiotics determined by broth microdilution and E-test.

Antibiotic	Susceptibility Criteria ^1^	Isolate 234509^T^	Isolate 335427^T^
Penicillin ^2^	ND	0.25	0.25
Amoxicillin ^2^	ND	0.38	0.75
Amoxicillin-clavulanic acid	≤8/4	<2/1	16/8
Ceftriaxone	≤8	<4	<4
Cefepime	≤8	<1	2
Imipenem	≤4	<2	<2
Clarithromycin	≤2	2	4
Doxycycline	≤1	<0.12	<0.12
Minocycline	≤1	<1	<1
Trimethoprim/sulfamethoxazole	≤2/38	<0.25/4.75	<0.25/4.75
Ciprofloxacin	≤1	>4	1
Levofloxacin	≤1	4	0.5
Tobramycin	≤4	<1	<1
Amikacin	≤8	<1	<1
Tigecycline	ND	0.06	0.25
Linezolid	≤8	<1	<1

^1^ According to CLSI criteria. ND: not defined. ^2^ CMI (µg/mL) determined by E-test

**Table 4:** Antimicrobial gene resistance genes in the genomes of isolates 335427^T^ and 234509^T^.

Isolate	Gene	AMR Gene Family	Drug Class	Resistance Mechanism	Identity of Matching Region (%)
**335427^T^**					
	*c*	small multidrug resistance (SMR) antibiotic efflux pump	aminoglycoside antibiotic, tetracycline antibiotic, phenicol antibiotic	antibiotic efflux	100
	*tet*44	tetracycline-resistant ribosomal protection protein	tetracycline	target protection	
	*lpe*	resistance-nodulation-cell division (RND) antibiotic efflux pump	-	efflux	100
	*van*HM	glycopeptide antibiotic	-	target alteration	100
**234509^T^**					
	-	EmrB/QacA subfamily drug resistance transporter	-		85.9
	-	MurA regulator CwlM implicated in cell wall metabolism and antibiotic tolerance	-		99.2

## Supplementary Materials

The following are available online at https://www.mdpi.com/2076-2607/8/10/1517/s1, Figure S1: Two-dimensional TLC plates of polar lipids extracted from strains 335427_T_ and DSM 44668_T_ sprayed using molybdatophosphoric acid (Sigma P1518). AGPL, aminoglycophospholipid; DPG, diphosphatidylglycerol; GPL, glycophospholipid;L; L, lipid; PE, phosphatidylethanolamine. Solvent 1: chloroform: methanol: distilled water (65:25:4 v/v/v); Solvent 2: chloroform: glacial acetic acid: methanol: distilled water (80:12:15:4 v/v/v/v), Table S1: Fatty acid profiles of isolates 335427_T_ and 234509_T_ and their closest phylogenetic neighbours. Only fatty acids more than 4% are listed, Table S2: Experimentally verified virulence genes on human and Mus musculus present in the genome sequences of isolates 335427_T_ and 234509_T_.
