# Supplementary material for: Genomic Virulence Features of Two Novel Species Nocardia barduliensis sp. nov. and Nocardia gipuzkoensis sp. nov., Isolated from Patients with Chronic Pulmonary Diseases
Source: Microorganisms. 2020 Oct 1;8(10):1517. doi: 10.3390/microorganisms8101517 (PMC7600791; doi:10.3390/microorganisms8101517)
Supplement: Supplementary file 1 [file microorganisms-08-01517-s001.pdf]

microorganisms

MDPI

Supplementary Material

Genomic Virulence Features of two novel species, *Nocardia barduliensis* sp. nov. and *Nocardia gipuzkoensis* sp. nov., isolated from patients with chronic pulmonary diseases

Imen Nouioui [1,*], Carlos Cortés-Albayay [2], Meina Neumann-Schaal [1], Diego Vicente [3], Gustavo Cilla [3], Hans-Peter Klenk [4], Jose María Marimón [3], Maria Ercibengoa [3,*]

[1] Leibniz Institute DSMZ–German Collection of Microorganisms and Cell Cultures, Braunschweig, Germany; imen.nouioui@dsmz.de; meina.neumann-schaal@dsmz.de

[2] Laboratory of Microbial Complexity and Functional Ecology, Antofagasta Institute, University of Antofagasta, Antofagasta, Chile; c.j.cortesbt@gmail.com;

[3] Biodonostia, Infectious Diseases Area, Respiratory Infection and Antimicrobial Resistance Group; Osakidetza Basque Health Service, Donostialdea Integrated Health Organisation, Microbiology Department, 20014 San Sebastian, Spain; diego.vicenteanza@osakidetza.eus; carlosgustavosantiago.cillaeguiluz@osakidetza.eus; josemaria.marimonortizdez@osakidetza.eus; maria.ercibengoaarana@osakidetza.eus (M.E)

[4] School of Natural and Environmental Sciences, Newcastle University, NE1 7RU Newcastle upon Tyne, United Kingdom; hans-peter.klenk@newcastle.ac.uk

* Correspondence: imen.nouioui@dsmz.de (I.N); maria.ercibengoaarana@osakidetza.eus (M.E)

Received: date; Accepted: date; Published: 1 October 2020

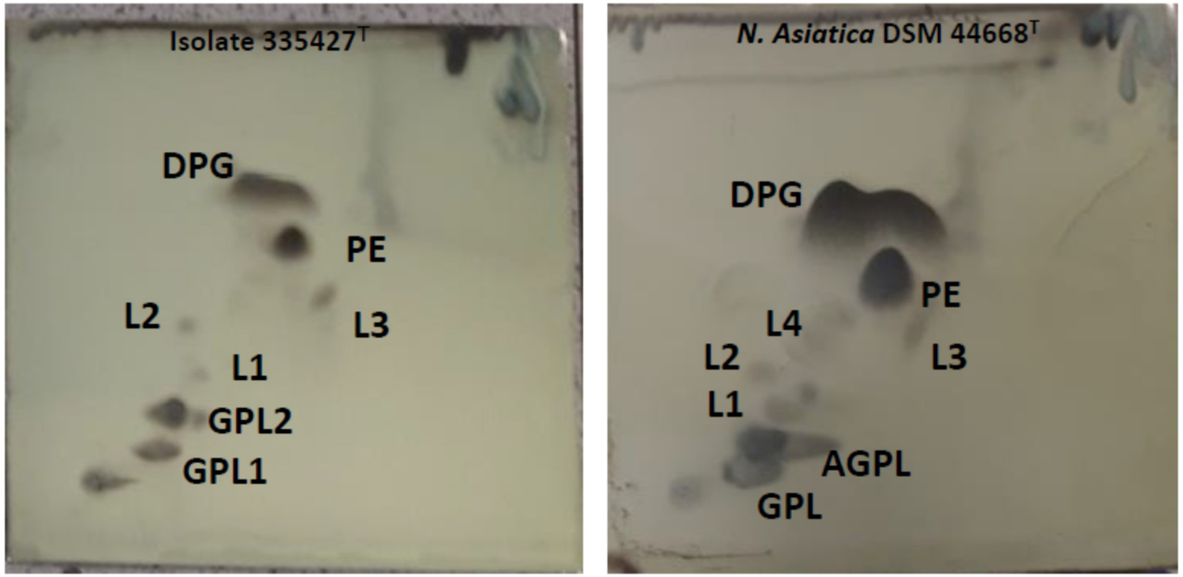

Figure S1. Two-dimensional TLC plates of polar lipids extracted from strains 335427^T and DSM 44668^T sprayed using molybdatophosphoric acid (Sigma P1518). AGPL, aminoglycophospholipid; DPG, diphosphatidylglycerol; GPL, glycophospholipid;L, lipid; PE, phosphatidylethanolamine. Solvent 1: chloroform: methanol: distilled water (65:25:4 v/v/v); Solvent 2: chloroform: glacial acetic acid: methanol: distilled water (80:12:15:4 v/v/v/v)

Table S1. Fatty acid profiles of isolates 335427^T and 234509^T and their closest phylogenetic neighbors. Only fatty acids more than 4% are listed.

	% of total composition			
**Fatty acids**	**Isolate 335427^T**	***N. asiatica* DSM 44668^T**	**Isolate 234509^T**	***N. abscessus* DSM 44432^T**
Summed feature 3 ($C_{16:1}$ 7c/$C_{16:1}\omega_6$c)	14.7	17.8	4.5	12.9
$C_{16:0}$	21.4	43.6	10.0	33.8
$C_{16:1}$ ω9c	-	-	6.2	-
$C_{18:0}$	25.4	-	-	9.5
$C_{18:0}$ 10 methyl	15.0	16.5	-	10.9
$C_{18:0}$ 1 ω7c	-	-	4.2	-
$C_{18:1}$ ω9c	16.6	15.6	70.0	24.0
$C_{19:0}$ *anteiso*	6.00	-	-	-

Table S2. Experimentally verified virulence genes on human and *Mus musculus* present in the genome sequences of isolates 335427ᵀ and 234509ᵀ.

Pathogenic gene	Function	Isolate		Isolate	
		Identity (%)	E-value	Identity (%)	E-value
*hrp*1 (*Rv*2626c)	hypoxic response protein 1	50	6,00E-45	50,3	6E-46
erm (37) (*Rv*1988)	Probable 23S rRNA methyltransferase Erm (37)	35,7	2E-23	35,2	8E-18
*anxa*1	Immune response	24,5	2,00E-05	24,2	1,00E-16
*stf*3	PAPS-dependent sulfotransferase Stf3	28,02	4,00E-18	67,7	5E-130
*dos*R (*dev*R)	Two-Component Regulatory Systems	79,9	R2-123	79,4	2E-122
*tcr*X	Probable transcriptional regulatory protein TcrX	54,9	2E-83	54,5	2E-88
*tcr*Y	Probable sensor histidine kinase TcrY	43,4	2E-74	43,2	5,00E-73
*trc*S	Two component sensor histidine kinase TrcS	39,5	1E-39	41,8	2,00E-54
*kdp*D	Sensor protein KdpD	31,1	2E-23	30,1	2,00E-11
*kdp*E	Transcriptional regulatory protein KdpE	41,3	1E-54	80,8	2,00E-136

*Rv*3213c	Possible SOJ/para-related protein	43,8	7E-67	43,8	7,00E-67
*pep*N	Probable aminopeptidase N PepN (Lysyl aminopeptidase) (LYS-AP) (Alanine aminopeptidase)	73,1	0E+00	72,7	0
*Rv*1773c	Probable transcriptional regulatory protein	30,8	9E-19	32,6	5,00E-20
*uma*A1	S-adenosylmethionine-dependent methyltransferase UmaA	33,3	3E-41	33	1,00E-41
*alk*B (Rv3252c)	Probable transmembrane alkane 1-monooxygenase AlkB (Alkane 1-hydroxylase) (Lauric acid omega-hydroxylase) (Omega-hydroxylase) (Fatty acid omega-hydroxylase) (Alkane hydroxylase-rubredoxin)	69	0	68	0
*cma*A2	Cyclopropane mycolic acid synthase 2	32,6	5E-42	33,1	2,00E-39
*fad*B4	Probable NADPH quinone oxidoreductase FadB4 (NADPH:quinone reductase) (zeta-crystallin)	66,3	1E-160	67	4,00E-161
*pkn*H	Serine/threonine-protein kinase PknH	60	1,00E-104	59,2	2,00E-103
*hsp*X	Alpha-crystallin	39,2	3,00E-18	38,2	2,00E-17
*Rv*3167c	Probable transcriptional regulatory protein	35,8	2,00E-23	37,2	9,00E-39

	(Probably TetR-family)				
*glp*X	Fructose-1,6-bisphosphatase class 2	81,5	0E+00	82	0
tap (*Rv*1258c)	Multidrug efflux pump Tap	26,5	1E-06	26	3,00E-10
*mmp*L11	transmembrane transport protein	30	6E-74	32	9,00E-72
*esp*L	ESX-1 secretion-associated protein EspL	38,4	5E-04	34,3	1,00E-05
*ppk*2 (Rv3232c)	Polyphosphate:GDP phosphotransferase	29,8	4,00E-31	32	4,00E-32
*Rv*0104	Uncharacterized protein Rv0104	25,6	2,00E-06	26,2	1,00E-06
*Rv*3404c	dTDP-4-amino-4,6-dideoxyglucose formyltransferase	31,4	1,00E-12	31,5	8,00E-13
*mt*3774 (*Ccs*X)	Thioredoxin-related protein	41	6E-44	40,2	5,00E-45
*cnp*B (cpdA)	3',5'-cyclic adenosine monophosphate phosphodiesterase CpdA	40,5	7,00E-59	41,5	6,00E-63
*lpq*S	Lipoprotein LpqS	34,2	7E-07	28,1	1,00E-06
*ppt*T	Phosphopantetheinyl transferase PptT (CoA:APO-[ACP]pantetheinephosphotransferase) (CoA:APO-[acyl-carrier protein]pantetheinephosphotransferase)	56,7	8E-85	55,4	7,00E-82

*Rv*0574c	Probable polyglutamine synthesis accessory protein Rv0574c	63	2,00E-84	60,2	6,00E-153

© 2020 by the authors. Licensee MDPI, Basel, Switzerland. This article is an open access article distributed under the terms and conditions of the Creative Commons Attribution (CC BY) license (http://creativecommons.org/licenses/by/4.0/).
